# The Influence of the Atmosphere, More Especially the Atmosphere of the British Isles, on the Health and Functions of the Human Frame; Embracing Observations on the Nature, Treatment, and Prevention of the Principal Diseases Resulting from Sudden Atmospherical Transitions. &c. &c.

**Published:** 1817-12

**Authors:** 


*The Influence of the Atmosphere, more especially the Atmosphere of the Bristish Isles, on the Health and Functions of the Human Frame; embracing Observations on the Nature, Treatment, and Prevention of the principal Diseases resulting from sudden Atmospherical Transitions. &c. &c.* By JAMES JOHNSON, M.D. Author of the Influence of Tropical Climates on European Constitutions; and one of the Editors of the Medico-Chirurgical Journal and Review. One Vol. Octavo, closely printed. pp. 294.

As it would be unnatural to censure and indecent to praise the work of our Colleague, we shall do little more than announce the publication, leaving it to our contemporaries to sit in judgement on its merits and imperfections. The following is Dr. Johnson's concise Preface:

" One part of the title page of the following Essay may induce some of those, who read no farther, to prejudge that the author has travelled from the direct line of his profession, and addressed himself to *general* readers. Were it even so, he does not think that he need be *ashamed*, however he might be *afraid*, to tread in the steps of a Hufeland and a Beddoes.* The case, however, is

---

* *Vide* Hufeland's " Art de prolonger la Vie," and Beddoes' " Hygeia."

otherwise. The author has in this, as in his Essay on 'The Influence of Tropical Climates on European Constitutions," left open to the general reader all that could be *useful*, and, consequently, all that can be *necessary*, for him to know; without, for a moment, descending from the dignity of a philosophical discussion, or forgetting that he was writing under the immediate eye of that *professional tribunal*, whose approbation *alone* he is anxious to merit—whose censure he is solicitous to avert.

"But as the execution of a work can only be estimated by the perusal, and its value appreciated by the application of its principles to practice; and as TIME very accurately adjusts the index of merit on the scale of literary rank, uninfluenced by a sounding title, a promising preface, or a *well spread* table of contents, the author does not deem it necessary to say much more, and he hopes he could not say much less.

"In respect to the Appendix on GOUT, the author conceives, that he has done the medical republic of his native soil some service, by translating and condensing a valuable *octavo volume* into eighty-one pages, without leaving behind a single useful fact, or ingenious observation. If he be not egregiously mistaken, this part of the work will merit and excite the attention of the medical world.

"As to the Appendix on Rheumatism, since the author is more personally concerned in it, he must necessarily speak of it with greater reserve. But he flatters himself, that it will not derogate from that rank which the *Naval Medical Corps* has of late years maintained, under great disadvantages, in the estimation of the profession at large—a corps that may fearlessly say, with their own immortal Falconer—

> Approach ye learn'd, and judge if we depart,
> Unequal, from the precepts of our Art.

"Whatever may be the demerits of the work, in other respects, it is, at least, free from one—the MEGA BIBLION evil. Under the genial influence of a modern typographical atmosphere, this volume would certainly have attained a stature and corpulence far beyond the middle size. It is to be hoped, however, that although the form be *slender*, and the features *small*, yet that the constitution is sound—and that it will long preserve a vigorous state of the *circulation and excitability*." Preface.

The work is dedicated in a very plain and philosophical inscription to the Duke of Clarence, by permission of the noble Duke; and from a note at the end of the preface, it appears that "nearly a fourth of the whole edition has already been ordered in Portsmouth and Portsea alone." This is somewhat unusual, and not a little flattering, considering how seldom a prophet is honoured in his own country!

The leading features of discussion in the *first* part of the work may be collected from the following passage:

"1*st*. The climate of England is remarkably variable; the mean temperature being about 52° Fahrenheit.

"2*ndly*. Sudden atmospherical transitions are very prejudicial to health.

"3*rdly*. The transitions are injurious to the constitution in proportion as the temperature has been much above or much below the medium heat of the time and place previously; and also in proportion to the length of time it continued at the opposite extreme: because the operation of heat predisposes the human frame to be more easily injured by sudden transitions to cold, and *vice versa*.

"4*thly*. Atmospherical impressions are primarily made on the skin and lungs.

"5*thly*. There is an intimate sympathy between the skin and lungs, which becomes the medium whereby atmospherical transitions occasion or increase the extensive class of *pulmonic* complaints, from a common catarrh to a confirmed phthisis. This class may be termed the national or climatorial disease, in the same manner as derangements of the *biliary* system form the most numerous class in tropical climates.

"6*thly*. The sympathy between the skin and stomach (cutaneo-gastric) leads to the production or aggravation of many complaints in the functions of the latter organ, from äerial vicissitudes acting on the surface.

"7*thly*. The *cutaneo-intestinal* sympathy, or consent between the skin and intestines, occasions derangements of the bowels in the same way.

"8*thly*. The *cutaneo-renal* sympathy, or consent between the surface and the kidnies, accounts for the vicarious increase of urine, when the pores of the skin are constricted, and the perspiration diminished.

"9*thly*. The *cutaneo-hepatic* sympathy, or consent between the functions of the skin and liver, accounts for the mode in which atmospherical impressions on the surface occasion or increase hepatic derangements, which have been too often mistaken for, and misnomered—nervous, dyspeptic, and hypochondriacal.

"10*thly*. The interior organs themselves, when once affected through sympathy with the surface, sympathize unequivocally with one another, producing various anomalous and complicated symptoms.

"11*thly*. Aerial vicissitudes produce scrofula, the taint or predisposition to which is transmitted from parent to progeny.

"It may be observed here, that if the various organs above-mentioned, sympathize so sensibly with the surface; so also do the skin and its functions sympathize, in turn, with them. Rarely, if ever, are the skin and perspiration natural, when any derangement is going forward in the structure or functions of the aforesaid viscera." p. 14, 15.

The principal diseases which are treated of, in detail, as resulting from the influence of climate, are, pneumonia, phthisis, gastritis, enteritis, dysentery, cholera, biliary derangements, scrofula, fever, gout, rheumatism.

The division of the work entitled HYGEIA, is subdivided into nine sections; "on the air we breathe, the food we eat, the fluids we drink, the exercise we use, the clothing we wear; ablutions, including the cold and warm bath; the passions, sleep, medicine."

The Appendix, on GOUT, is a condensed translation, with many Notes, of Dr. Guilbert's new work on that subject, and which is a transcript of the learned and valuable article on gout, in the last volume of the "Dictionaire des Sciences Medicales."

In concluding the translation, Dr. Johnson remarks, "I have thus condensed an octavo volume into 81 pages, without leaving a single fact or ingenious observation behind. The labour has not been inconsiderable; and to one less accustomed to this species of composition, the task would have been extremely difficult. I may venture to assert, that in no equal number of pages, in the English, or in any other language, will *one half of the quantum* of valuable matter be found, as is presented in this Appendix." p. 240.

The second Appendix is on Acute and Chronic Rheumatism, wherein the author acknowledges his obligations to Dr. Dickson of Clifton, Dr. M'Arthur of Deal, Drs. Porter, Henderson, and Felix, of Bristol, Mr. Sheppard of Witney, Dr. Archibald Robertson of Edinburgh, and several other naval medical friends, for important communications.*

The third Appendix is on the Nitro-Muriatic Acid Bath, in bilious derangements.

To use Dr. Johnson's own words — "we hope we need not say more; and we think we could not say much less."

---

* In this Appendix, Dr. Johnson has given the only complete history, yet published, of cardiac diseases resulting from metastasis of rheumatic inflammation to the central organ of the circulation.

